# Respiratory Gating during Stereotactic Body Radiotherapy for Lung Cancer Reduces Tumor Position Variability

**DOI:** 10.1371/journal.pone.0112824

**Published:** 2014-11-07

**Authors:** Tetsuo Saito, Tomohiko Matsuyama, Ryo Toya, Yoshiyuki Fukugawa, Takamasa Toyofuku, Akiko Semba, Natsuo Oya

**Affiliations:** Department of Radiation Oncology, Kumamoto University Hospital, Kumamoto, Japan; Northwestern University Feinberg School of Medicine, United States of America

## Abstract

**Purpose:**

We evaluated the effects of respiratory gating on treatment accuracy in lung cancer patients undergoing lung stereotactic body radiotherapy by using electronic portal imaging device (EPID) images.

**Materials and Methods:**

Our study population consisted of 30 lung cancer patients treated with stereotactic body radiotherapy (48 Gy/4 fractions/4 to 9 days). Of these, 14 were treated with- (group A) and 16 without gating (group B); typically the patients whose tumors showed three-dimensional respiratory motion ≧5 mm were selected for gating. Tumor respiratory motion was estimated using four-dimensional computed tomography images acquired during treatment simulation. Tumor position variability during all treatment sessions was assessed by measuring the standard deviation (SD) and range of tumor displacement on EPID images. The two groups were compared for tumor respiratory motion and position variability using the Mann-Whitney U test.

**Results:**

The median three-dimensional tumor motion during simulation was greater in group A than group B (9 mm, range 3–30 mm vs. 2 mm, range 0–4 mm; p<0.001). In groups A and B the median SD of the tumor position was 1.1 mm and 0.9 mm in the craniocaudal- (p = 0.24) and 0.7 mm and 0.6 mm in the mediolateral direction (p = 0.89), respectively. The median range of the tumor position was 4.0 mm and 3.0 mm in the craniocaudal- (p = 0.21) and 2.0 mm and 1.5 mm in the mediolateral direction (p = 0.20), respectively.

**Conclusions:**

Although patients treated with respiratory gating exhibited greater respiratory tumor motion during treatment simulation, tumor position variability in the EPID images was low and comparable to patients treated without gating. This demonstrates the benefit of respiratory gating.

## Introduction

The advent of four-dimensional computed tomography (4D-CT) planning and of methods that mitigate the effects of tumor respiratory motion during irradiation has the potential to improve the therapeutic ratio of radiotherapy for lung cancer [1]–[4]. Respiratory gating is a non-invasive technique for addressing the problem posed by the respiratory motion of tumors including lung cancers [5]–[10]. Based on the results of our earlier simulation studies that compared respiratory gating techniques [11], [12] we used phase-based gating in patients with lung cancer to set the gating window to cover a 30% duty cycle around end-expiration. As our earlier investigations were treatment simulation studies we set out to confirm the accuracy of treatment delivery in patients we treated with respiratory gating and checked the validity of our selection criteria to identify patients eligible for respiratory gating [13]. Although others [7], [8] who applied analysis of treatment planning suggested that respiratory gating offers theoretical benefits, this issue remained to be examined in the clinical setting.

The electronic portal imaging device (EPID) makes it possible to determine whether gating reduces the variability in the tumor position and the continuous acquisition of portal images with the EPID in cine mode has been used for treatment verification [14]–[16]. In the current study we used EPID images to assess the effect of respiratory gating on treatment accuracy. Prior studies have reported that lung tumors with greater respiratory motion during treatment simulation tend to show greater position variability during treatment than did static tumors [17]–[19]. Because we tended to select patients with mobile tumors for respiratory gating, these tumors might show large position variability without gating. If the mobile tumors treated with gating show low position variability during the gated phases, this should indicate the benefit of respiratory gating. In this study, we compared tumor respiratory motion and position variability between the patient groups treated with and without respiratory gating.

## Materials and Methods

### Patients

Between October 2008 and May 2013 we treated 62 consecutive patients with 69 primary and metastatic lung cancers with stereotactic body radiotherapy. The current study population is comprised of 30 of these patients whose 30 tumors were clearly visible on EPID images acquired during their treatment sessions. We subjected 14 patients to respiratory gating (group A); the other 16 were treated without gating (group B). To identify patients eligible for gating we primarily considered tumor motion during 4D-CT simulation treatments. Typically we chose patients whose tumors showed three-dimensional respiratory motion ≧5 mm [13]. We determined not to use respiratory gating for the patients with irregular breathing (frequency and/or amplitude) by evaluating the respiratory curves recorded at 4D-CT data acquisition. Lung irradiation volume was another factor we considered in selecting patients for gating. The average tumor diameter was 20 mm (range 9–40 mm) in group A and 21 mm (range 10–36 mm) in group B. Of the 14 tumors in group A, 2 were located in the upper lobe, one in the middle lobe, and 11 in the lower lobe; 15 group B tumors were in the upper lobe and one was in the lower lobe. All patients gave their written informed consent for use of their data for research purposes before treatment. Tumor characteristics are shown in Table S1. The retrospective data analysis in this study was approved by the institutional review board at the Kumamoto University (No. 790).

### Treatment

We used a vacuum-formed cushion (ESFORM, Engineering System, Matsumoto, Japan) for body immobilization. The CT scanner used for data acquisition was a GE LightSpeed RT (GE Medical Systems, Waukesha, WI, USA) instrument. Details of our 4D-CT procedure are described elsewhere [11]. An external respiratory monitor system (Real-time Position Management System, Varian Medical Systems, Palo Alto, CA, USA) recorded respiratory motion in temporal correlation with CT scan acquisition. For target delineation and treatment planning we used a treatment planning system (XiO, Elekta, Stockholm, Sweden). The clinical target volume (CTV) was contoured to include the gross tumor volume and the microscopic tumor extension. Individual CTVs in selected phases were combined to form composite CTVs and considered the internal target volume (ITV). The planning target volume (PTV) was defined as an expansion of the ITV with a 5- to 8- mm margin in the craniocaudal (CC) direction and a 5-mm margin in the other directions. The PTV margin in the CC direction was determined individually according to the tumor respiratory motion on 4D-CT images. Typically we added a 5-mm leaf margin to the PTV.

For radiotherapy we used a Clinac iX instrument (Varian Medical Systems); the dose rate was 600 MU/min. Stereotactic body radiotherapy was delivered via 6 coplanar and non-coplanar static beams using 6 MV photons. The prescribed dose was 48 Gy delivered to the isocenter in 4 fractions in the course of 4 to 9 days (median 5 days). The patient setup was corrected by using an on-board kilovoltage cone-beam CT scanner; the images were acquired under free-breathing conditions. In group A we used phase-based gating around end-expiration and set a length of 30% of a full respiratory cycle as the gating window. During gated treatment the external respiratory monitor system (Varian Medical Systems) synchronized the treatment with the patient's respiratory cycle.

### Tumor motion during simulation

To evaluate three-dimensional tumor respiratory motion we used 4D-CT images acquired during treatment simulations. One radiation oncologist (T.S.) delineated the CTVs on CT images of two extreme respiratory phases. We measured the distance from the end-inspiration CTV centroid to the end-expiration CTV centroid using a treatment planning system (XiO, Elekta, Stockholm, Sweden).

### Image acquisition during treatment

For cine image acquisition during treatment we used an amorphous silicon EPID (aS1000, Varian Medical Systems) mounted on the Clinac iX. The EPID had an active area of 40×30 cm that contained 1024×768 pixels. The EPID images were acquired at 7.5 frames per sec (20 frames per one EPID image). We routinely acquired EPID cine images of all treatment ports in all treatment sessions. In patients treated before September 2010 we were unable to obtain EPID cine images of the same port as was used for portal image acquisition before treatment (the gantry angle was 180° in most patients), and therefore, we did not have EPID images of all sessions.

### Tumor position variability during treatment

**Figure 1:**
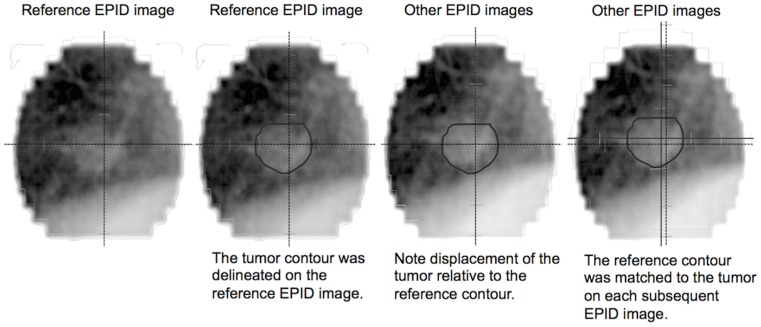
Tumor displacement measured with reference to the first EPID image acquired at the first session. EPID  =  electronic portal imaging device.

**Figure 2:**
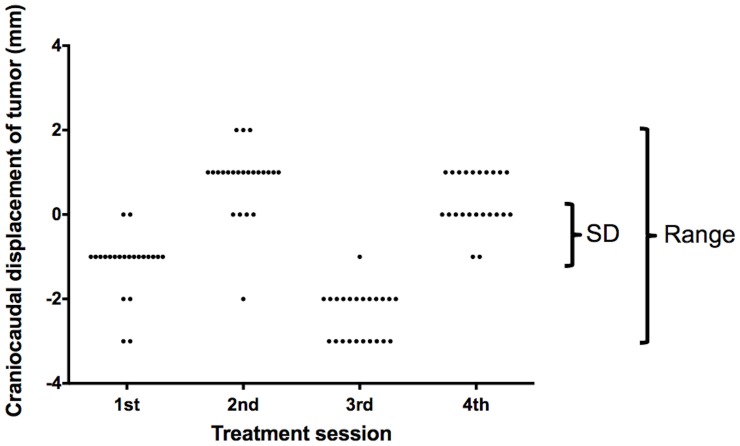
Variability assessment for each patient. Variability was assessed by determining the standard deviation (SD) and range of the tumor position during all sessions.

Tumor position variability was evaluated by the radiation oncologist (T.S.) To identify the tumor position relative to a reference image we used registration software (offline review, Varian Medical Systems). The software provides an integer displacement value. We evaluated only EPID images obtained at a gantry angle of 180°. Figure 1 shows our procedure for measuring the tumor position. In all treatment sessions we used the first EPID image acquired at the first session as the reference for subsequent measurements on all other EPID images. We first delineated the tumor on this reference EPID image and then used its contour for measuring tumor displacement on the other EPID images. By matching the contour to the tumor image on each EPID image we were able to measure the displacement of the tumor position relative to the reference EPID image (Figure 1). Tumor displacement was measured in the CC and the mediolateral (ML) direction. EPID images acquired during 2 to 4 (average 3.4) treatment sessions per patient were used for variability assessments. We analyzed a total of 1,168 EPID images acquired in the course of 101 sessions. In each patient we assessed variability in the tumor position by measuring the standard deviation (SD) and the range of the tumor position during all sessions (Figure 2).

### Inter-observer variation

To assess inter-observer variations in the measured tumor position we used the second and last EPID images from each session (202 images acquired in 101 sessions). The tumor position relative to the first EPID image obtained at the first session was independently measured by 2 radiation oncologists (T.S. and T.M.) and the average and the SD of the difference between their measurements were calculated. Inter-observer variation was assessed in the CC and ML direction.

### Statistical analysis

We used the Mann-Whitney U test to compare tumor respiratory motion and position variability in groups A and B. The Wilcoxon signed-rank test was used to compare the tumor positions measured by the 2 radiation oncologists. All statistical tests were performed with GraphPad Prism 6 software (GraphPad Software Inc., San Diego, CA, USA). Differences of p<0.05 were considered statistically significant.

## Results

### Inter-observer variation

Inter-observer variations in the tumor localization were assessed by measuring the difference in the tumor position recorded by the two observers. For a total of 202 observations, the average (SD) difference between them was 0.3 mm (0.8 mm) in the CC direction and 0.3 mm (0.8 mm) in the ML direction (Table S2). The tumor positions measured by the 2 radiation oncologists were statistically significantly different in the CC direction (p<0.001) and in the ML direction (p<0.001).

### Tumor motion during treatment simulation

**Figure 3:**
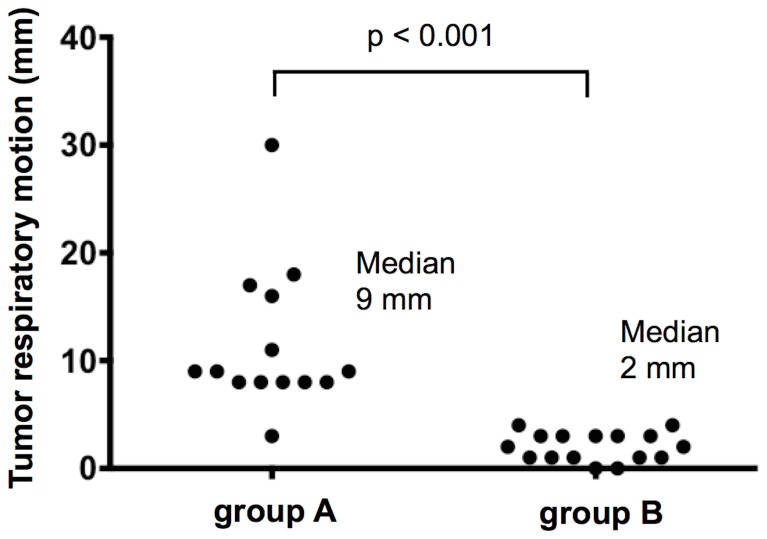
Three-dimensional tumor respiratory motion during treatment simulation evaluated with four-dimensional CT.

Figure 3 shows the three-dimensional tumor respiratory motion measured during treatment simulation using 4D planning CT. The median tumor motion was greater in group A than B (9 mm, range 3–30 mm vs. 2 mm, range 0–4 mm) (p<0.001) (Table S1).

### Tumor position variability during treatment

**Figure 4:**
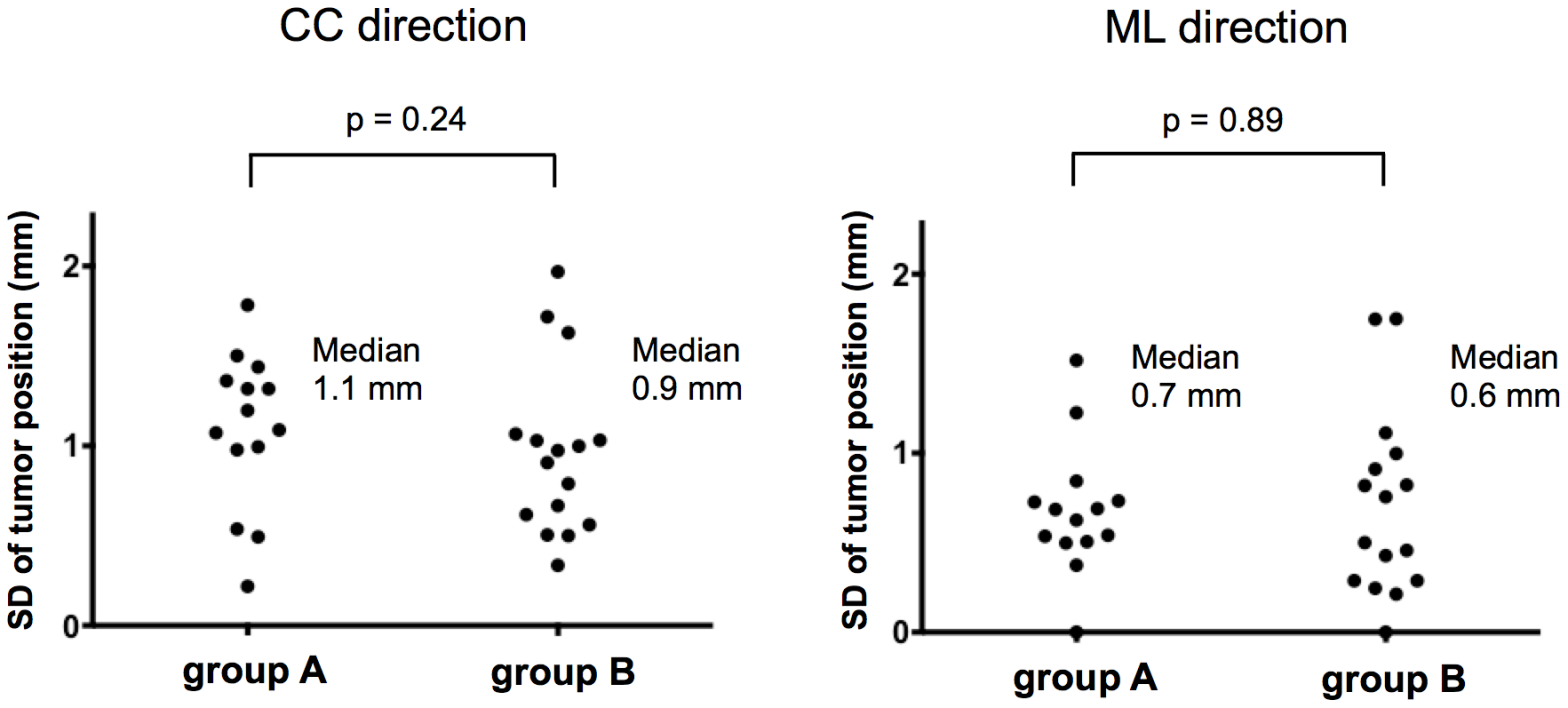
Standard deviation (SD) of the tumor position on electronic portal imaging device (EPID) images. Each point represents a single patient's SD of the tumor position during all treatment sessions. CC  =  craniocaudal; ML  =  mediolateral.

**Figure 5:**
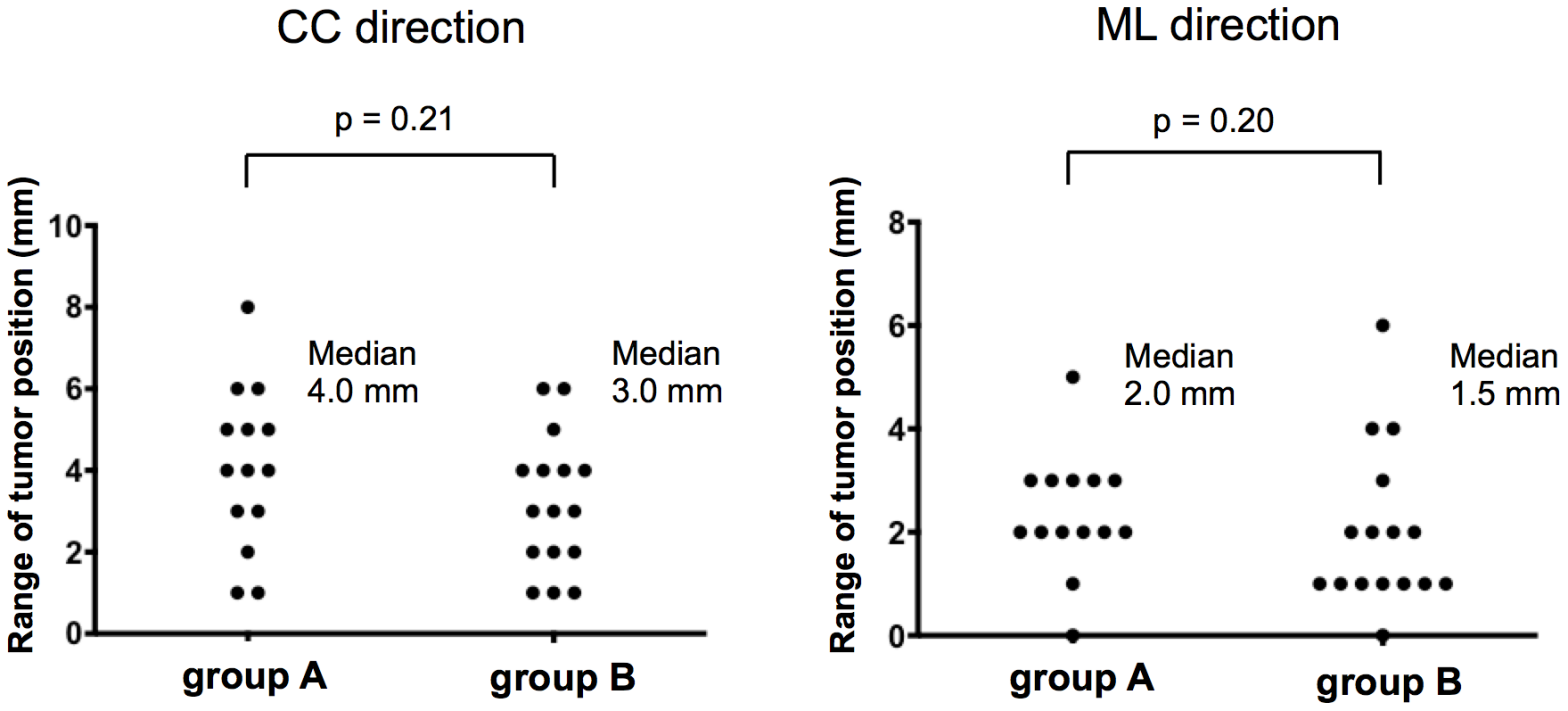
Range of the tumor position on electronic portal imaging device (EPID) images. Each point represents a single patient's range of the tumor position during all treatment sessions. CC  =  craniocaudal; ML  =  mediolateral.

For variability assessments we used EPID images acquired during treatment. The SD and the range of the tumor position during all treatment sessions were calculated for each patient (Figure 2) (Table S3, Table S4). Figure 4 and 5 show the SDs and the ranges of the tumor position, respectively, in the two patient groups. In group A and group B the median SD of the tumor position was 1.1 mm and 0.9 mm in the CC (p = 0.24) and 0.7 mm and 0.6 mm in the ML direction (p = 0.89), respectively. The median range of the tumor position was 4.0 mm and 3.0 mm in the CC (p = 0.21) and 2.0 mm and 1.5 mm in the ML direction (p = 0.20), in group A and B, respectively. The difference in variability between the two groups was not statistically significant. The range of tumor displacement in the CC direction did not exceed 6 mm in any but one patient who was subjected to respiratory gating and manifested a range of 8 mm. The range of tumor displacement in the ML direction was not more than 6 mm in any of the 30 patients.

## Discussion

Using EPID images we found that tumor position variability in our patients treated with and without respiratory gating was low and comparable to that reported in earlier studies [17], [20]–[22] (Table 1). Patients subjected to gating manifested greater respiratory motion during simulation, however, tumor position variability during treatment was comparable to patients treated without gating. Our findings, based on data acquired during the treatment of lung cancer patients, confirmed that respiratory gating was beneficial in reducing tumor position variability.

**Table 1:** Earlier reports on the variability of the lung tumor position evaluated on EPID images.

Authors (year) [ref]	Number of patients	Respiratory motion during simulation	Respiratory gating	Intra/interfractional variability	Variability assessed by SD^c^	Variability assessed by range^d^
Ford EC (2002) [20]	2	6.9 mm	yes	intrafractional^a^	2.6–5.7 mm (range)	-
Gaede S (2008) [21]	4	-	yes	intrafractional^a^	0.94 mm (not specified)	-
	1	-	no		2.1 mm (not specified)	-
Spoelstra FOB (2008) [22]	11	5.0–19.5 mm	yes	Interfractional^b^	1.3–2.1 mm (range)	-
Ueda (2012) [17]	28	3.1 mm	no	intrafractional^a^	-	4.0 mm (mean)
Present study	14	9 mm	yes	Interfractional^b^	1.1 mm (median)	4.0 mm (median)
	16	2 mm	no		0.9 mm (median)	3.0 mm (median)

EPID  =  electronic portal imaging device, SD  =  standard deviation.

a Tumor position variability during one treatment session.

b Tumor position variability during more than one treatment session.

c SD of the tumor position in the craniocaudal direction.

d Range of the tumor position in the craniocaudal direction.

Our findings also confirm that the selection criteria we used to identify patients who may benefit from respiratory gating were appropriate. To achieve low variability, patients with highly mobile tumors should be excluded from the group treated without gating. Had our criteria been excessively conservative (i.e. no respiratory gating in some patients with mobile tumors), we would expect to find a large variability in the tumor position during treatment without gating. Therefore, the low variability in patients treated without gating suggests that our selection criteria appropriately excluded patients with highly mobile tumors. Based on our previously-reported treatment planning analysis [13] we consider tumors with three-dimensional respiratory motion ≧5 mm on simulation 4D-CT images eligible for respiratory gating. Regularity of breathing and the expected lung irradiation volume are additional factors to be considered in the selection of patients subjected to respiratory gating. Breathing irregularity may lead to inaccurate delineation of gating window ITV [23], compromising the validity of treatment planning in respiratory-gated radiotherapy. When the tumor is large and/or located in the lower lobe, the lung irradiation volume tends to be large, and risk of pulmonary toxicity should increase the necessity of respiratory gating.

The inter-observer difference with respect to tumor localization was small and validates the quantitative assessment of our study. Inter-observer differences in the evaluation of EPID images of lung cancer patients have been evaluated by others [22], [24], [25] who did not use fiducial markers. Spoelstra et al. [22] who examined inter-observer variations in the identification of internal structures on time-integrated electronic portal images found that the SD of the variation between two observers, for a total of 57 observations, was 0.7 and 0.8 mm in the ML and CC direction, respectively. Muirhead et al. [24] analyzed megavoltage cine-images from patients with locally advanced lung cancer; they reported that for two observers the mean difference in motion of tumor, hilar structure and carina was 0.41 mm, 0.63 mm, and 0.33 mm, respectively. The inter-observer differences in our study were comparable to these earlier studies. We observed that the tumor position measured by the 2 radiation oncologists was statistically significantly different in this relatively large sample (n = 202). However, this difference would not be clinically relevant because the absolute value of the difference between the two observers was substantially small.

The intra- and interfractional variability in the tumor position has been investigated [17], [20]–[22] (Table 1). The magnitude of intrafractional variability may reflect factors such as body immobilization and tumor respiratory motion. In addition, interfractional variability reflects errors in image guidance and interfractional variations in respiration; it can be larger than intrafractional variability [26]. For accurate treatment, respiratory gating should control not only respiratory tumor motion during a fraction but also variations in respiration between fractions. Our comprehensive analysis of all EPID images acquired during all treatment sessions suggests that respiratory gating reduces both intra- and interfractional variability in the position of lung tumors.

Lung tumors near the diaphragm has tended to show large respiratory motion [2], [13]. In our patients, of the 14 tumors subjected to respiratory gating (group A), 11 were located in the lower lobe while 15 of the 16 tumors treated without gating (group B) were in the upper lobe. This may explain the difference in tumor respiratory motion between the two groups and their comparable tumor position variability indicates that respiratory gating compensates for tumor mobility.

As a limitation of our study, it was comprised of 30 of 69 (43%) consecutively-treated lung tumors and included only tumors that were clearly visible on EPID images. Richter et al. [18] who analyzed the feasibility of markerless tracking of lung tumors reported that tumor visibility was sufficient in 47% of their EPID movies. In the study of Ueda et al.[17], 38% of EPID images were used for analysis of lung tumor motion without fiducial markers.

In earlier investigations the reported potential benefit of respiratory gating was primarily based on planning-based analyses [7], [8], [27]. Few studies evaluated the benefit of respiratory gating based on data acquired during radiotherapy. We investigated the variability in the location of lung tumors on EPID images from a relatively large number of patients. To our knowledge, this is the first comparison of lung cancer patients subjected to radiotherapy with and without respiratory gating. In earlier studies, tumor position variability was evaluated mainly in a single group of patients. Consequently, the effect of gating could not be assessed separately from other sources of variability [17], [20]–[22]. To better understand the effect of respiratory gating, we are continuing to collect data obtained in lung cancer patients treated with- and without respiratory gating.

## Conclusions

Using EPID image-based analysis we evaluated the effect of respiratory gating on treatment accuracy. Tumor position variability during treatment was comparable in patients treated without- and patients treated with respiratory gating, despite greater respiratory motion during simulation in the latter group. This observation confirms the benefit of respiratory gating. Based on our findings we suggest that proper patient selection and the appropriate use of respiratory gating facilitate the accurate delivery of treatment in patients with lung cancer.

## Supporting Information

Table S1 **Tumor characteristics and respiratory motion.**
(XLS)

Table S2 **Inter-observer variation in tumor localization.**
(XLS)

Table S3 **Tumor position variability during treatment in group A.**
(XLS)

Table S4 **Tumor position variability during treatment in group B.**
(XLS)
